# Phylogenetic affinities of the sequestrate genus *Rhodactina* (Boletaceae), with a new species, *R. rostratispora* from Thailand

**DOI:** 10.3897/mycokeys.29.22572

**Published:** 2018-01-25

**Authors:** Santhiti Vadthanarat, Olivier Raspé, Saisamorn Lumyong

**Affiliations:** 1 Department of Biology, Faculty of Science, Chiang Mai University, Chiang Mai, 50200, Thailand; 2 Botanic Garden Meise, Nieuwelaan 38, 1860 Meise, Belgium; 3 Fédération Wallonie–Bruxelles, Service général de l’Enseignement universitaire et de la Recherche scientifique, Rue A. Lavallée 1, 1080 Bruxelles, Belgium

**Keywords:** *atp*6, Boletales, Diversity, Leccinoideae, Phylogeny, Taxonomy

## Abstract

*Rhodactina* is a small sequestrate genus in Boletaceae with two described species, *R. himalayensis* and *R. incarnata*. Phylogenetic analyses of a three-gene dataset including *atp*6, *tef*1 and *rpb*2 of *Rhodactina* species along with selected Boletaceae species showed that all *Rhodactina* species formed a monophyletic clade, sister to the genera *Spongiforma* and *Borofutus* in subfamily Leccinoideae with high support. All of the taxa in the clade have a similar chemical reaction in which basidiospores turn purplish, purplish red to violet or violet grey when in contact with potassium hydroxide. The molecular analyses also showed that all *Rhodactina* specimens collected from Ubon Ratchathani province, northeastern Thailand, belong to a new species. Morphologically, the new species is different from others by having a markedly prominent hilar appendage and a terminal hilum on its basidiospores. Thus, the new species, *Rhodactina rostratispora*, is introduced with detailed macroscopic and microscopic descriptions and illustrations.

## Introduction

The genus *Rhodactina* Pegler & T.W.K. Young was first described in 1989 with *R. himalayensis* Pegler & T.W.K. Young, from northwestern India, as the type species. Typical characters of the genus are a whitish to pinkish puffball like basidiomata lacking both stipe and columella, violet brown to purple brown or pale pink to red hymenophore when mature, combined with purplish to purplish red, dextrinoid basidiospores with longitudinal ridges, lack of both clamp connections and cystidia. The genus was originally classified based on morphological characters in the family Gautieriaceae Zeller as the spore ornamentation was originally viewed as similar to the genera *Gautieria* Vittad and *Austrogautieria* E.L. Stewart & Trappe (Pegler and Young 1989). In 2006, the second species, *R. incarnata* Zhu L. Yang, Trappe & Lumyong was described and the known distribution of *R. himalayensis* was extended to Chiang Mai Province, northern Thailand. Based on the phylogenetic analyses of *atp*6 sequences, the genus was moved to the family Boletaceae Chevall (Yang et al. 2006). However, the phylogenetic affinities of *Rhodactina* within the Boletaceae remained unclear because of very limited taxon sampling. So, at present, there are only two described *Rhodactina* species, *R. himalayensis* and *R. incarnata* (http://www.indexfungorum.org/Names/Names.asp), both of which have been reported to occur in northern Thailand (Chandrasrikul et al. 2011).

Boletaceae diversity seems to be high in Thailand (Chandrasrikul et al. 2011), with several new species described in the last five years (Choeyklin et al. 2012, Halling et al. 2014, Neves et al. 2012, Raspé et al. 2016). During this survey of Boletaceae diversity in Thailand, several *Rhodactina* collections were made and their morphology and phylogenetic relationships were studied. Phylogenetic analyses were based on three genes: *atp*6, *tef*1 and *rpb*2, which have previously been justified as useful for phylogenetic analyses of Boletales (Kretzer and Bruns 1999, Binder and Hibbett 2006, Hosen et al. 2013, Li et al. 2014, Smith et al. 2015, Orihara et al. 2016, Raspé et al. 2016, Wu et al. 2016). Both morphology and phylogenetic analyses confirmed that all newly collected specimens belong to a new species in the genus *Rhodactina*. Thus, the third species of *Rhodactina*, found in Thailand, is described and its phylogenetic affinities are presented in this study.

## Materials and method

### Specimens collecting

The new *Rhodactina* specimens were collected and photographed from community forests in Trakan Phuet Phon district, Ubon Ratchathani province, northeastern Thailand, in the rainy season during 2015–2017. The specimens were wrapped using aluminium foil or kept in plastic boxes until return to the laboratory and described within 24 h. The specimens were dried in an electric drier at 45–50 °C. The examined specimens are deposited in the herbaria CMUB and BR (both listed in Index Herbariorum; Thiers, continuously updated).

### Morphological studies

The macroscopic description was based on detailed field notes and photos of basidiomata. Colour codes followed Kornerup and Wanscher (1978). Macrochemical reactions (colour reactions) of peridium, hymenophore and microscopic structures were determined using 5 % (w/v) aqueous potassium hydroxide, 28–30 % ammonium hydroxide or Melzer’s reagent. Microscopic structures were observed from dried specimens, rehydrated in 5% KOH or 1 % ammoniacal Congo red. For each collection, a minimum of 50 basidiospores and 20 basidia were randomly selected and measured at 1000× with a calibrated ocular micrometer using an Olympus CX31 microscope. Spore dimensions include ornamentation. The notation ‘(*n*/*m*/*p*)’ represents the number of basidiospores *n* measured from *m* basidiomata of *p* collections. Dimensions of microscopic structures are presented in the following format: (*a*–)*b*–*c*–*d* (–*e*), in which *c* represents the average, *b* the *5^th^* percentile, *d* the *95^th^* percentile and extreme values *a* and *e* are shown in parentheses. *Q*, the length/width ratio, is presented in the same format. Sections of the peridium surface were made radially and perpendicularly to the surface, halfway between the centre and the side of basidiomata. All microscopic features were drawn free hand using an Olympus Camera Lucida model U−DA fitted to the microscope cited above. For scanning electron microscopy (SEM), small fragments of dried hymenophore were mounted directly on to an SEM stub with double-sided tape. The samples were coated with gold for 60 seconds using SPI-Module Sputter Coater, examined and photographed at 15–20 kV with a FIB Quanta 200 3D scanning electron microscope (Thermo Fisher Scientific, United States).

### DNA isolation, PCR amplification and DNA sequencing

Genomic DNA was extracted from fresh tissue preserved in CTAB or about 10–15 mg of dried specimens using a CTAB isolation procedure adapted from Doyle and Doyle (1990). The genes *atp*6, *tef*1 and *rpb*2 were amplified by polymerase chain reaction (PCR) technique. For the amplification of *atp*6, ATP6-1M40F and ATP6-2Mprimers were used (Raspé et al. 2016), with the following PCR programme: 2 min at 95 °C; 5 cycles of 45 s at 95 °C, 60 s at 42 °C, 30 s at 72 °C; 35 cycles of 20 s at 95 °C, 30 s at 55 °C, 30 s+1 s/cycle at 72 °C; 3 min at 72 °C. The primers EF1-983F and EF1-2218R (Rehner and Buckley 2005) were used to amplify *tef*1 and bRPB2-6F and bRPB2-7.1R primers (Matheny 2005) were used to amplify *rpb*2. PCR products were purified by adding 1 U of Exonuclease I and 0.5 U FastAP Alkaline Phosphatase (Thermo Scientific, St. Leon-Rot, Germany) and incubated at 37 °C for 1 h, followed by inactivation at 80 °C for 15 min. Sequencing was performed by Macrogen Inc. (Korea and The Netherlands) with PCR primers, except for *atp*6, for which universal primers M13F-pUC(-40) and M13F(-20) were used; for *tef*1, additional sequencing was performed with the two internal primers, EF1-1577F and EF1-1567R (Rehner and Buckley 2005).

### Alignment and phylogeny inference

The sequences were assembled in GENEIOUS Pro v. 6.0.6 (Biomatters) and introns were removed prior to alignment based on the amino acid sequence of previously published sequences. All sequences, including sequences from GenBank, were aligned using MAFFT (Katoh and Standley 2013) on the server accessed at http://mafft.cbrc.jp/alignment/server/. Maximum Likelihood (ML) phylogenetic tree inference was performed using RAxML (Stamatakis 2006) on the CIPRES web server (RAxML-HPC2 on XSEDE; Miller et al. 2009). The phylogenetic tree was inferred by a single analysis with three partitions (one for each gene), using the GTRCAT model with 25 categories and three *Chalciporus* species were used as an outgroup. Statistical support of nodes was obtained with 1,000 bootstrap replicates.

## Results

### DNA analyses

**Figure 1.**
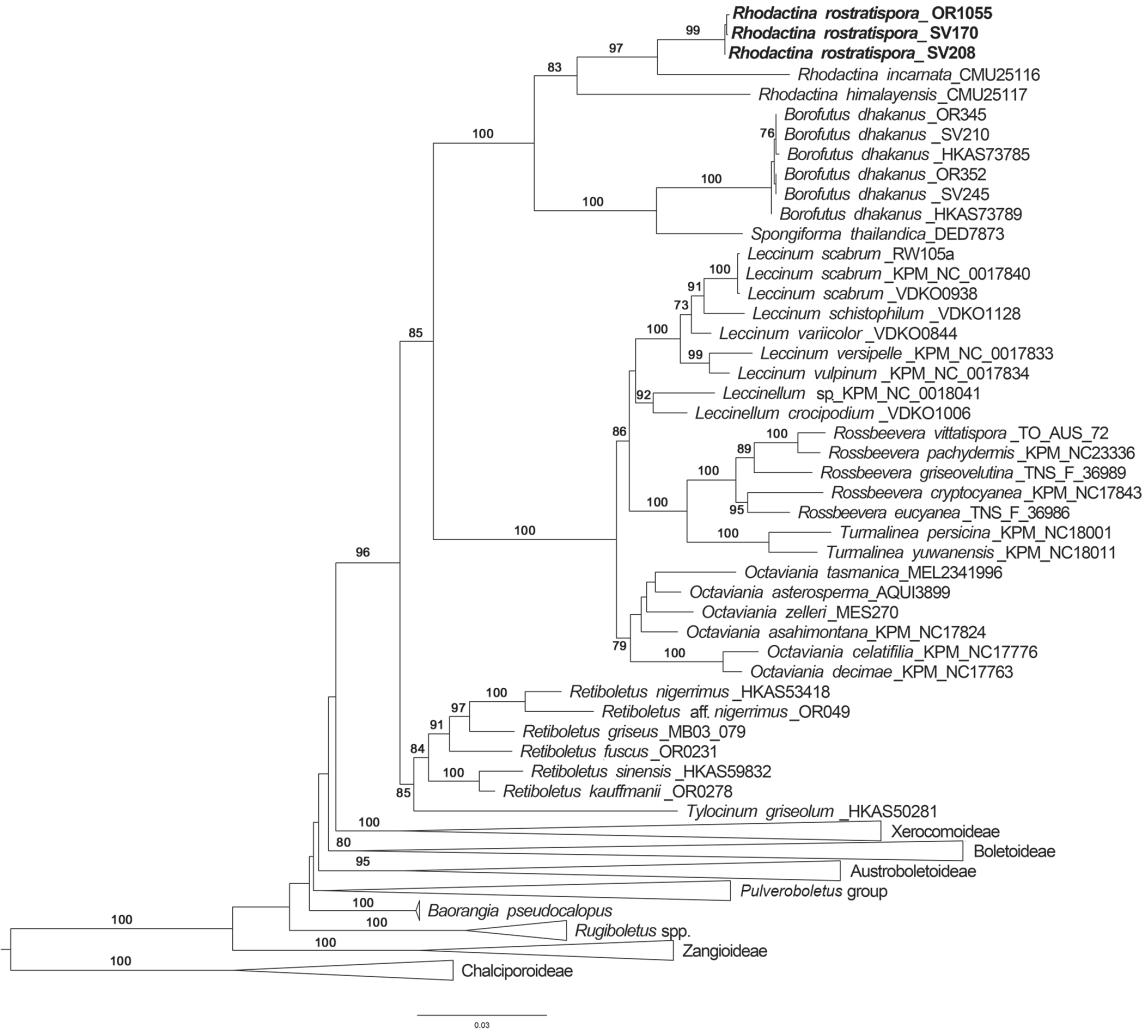
Maximum likelihood phylogenetic tree inferred from the three-gene dataset (*atp*6, *rpb*2, *tef*1), including *Rhodactina rostratispora* and selected Boletaceae. The three *Chalciporus* species were used as outgroup taxa. Most of the taxa not belonging to the subfamily Leccinoideae were collapsed into subfamilies or similar level clade (i.e. *Pulveroboletus* group). Bootstrap support values > 70% are shown above branches.

A total of 127 new sequences were generated and deposited in GenBank (Table 1). The alignment contained 157 taxa spread over the entire family Boletaceae and was 2429 characters long (TreeBase number 21933). The authors could not obtain *tef*1 and *rpb*2 sequences from *R. incarnata* (CMU25116) nor *rpb*2 sequence from *R. himalayensis* (CMU25117). The specimens were in relatively poor condition and genomic DNA was highly degraded. The 3-gene phylogram indicated that all selected collections of the new taxon *R. rostratispora* formed a monophyletic group with high bootstrap support sister to *R. incarnata* within the *Rhodactina* clade (Figure 1). The *Rhodactina* clade was sister to a clade composed of the genera *Spongiforma* Desjardin, Manfr. Binder, Roekring & Flegel and *Borofutus* Hosen & Zhu L. Yang, within the subfamily Leccinoideae G. Wu & Zhu L. Yang clade. Interestingly, the genera *Rhodactina*, *Spongiforma* and *Borofutus* formed a clade with 100% bootstrap support.

**Table 1.** List of collections used for DNA analyses, with origin, GenBank accession numbers and reference(s).

Species	Voucher	Origin	*atp*6	*tef*1	*rpb*2	References
*Afroboletus costatisporus*	ADK4644	Togo	KT823958	KT824024	KT823991	Raspé et al. 2016
*Aureoboletus catenarius*	HKAS54467	China	–	KT990711	KT990349	Wu et al. 2016
*Aureoboletus duplicatoporus*	HKAS50498	China	–	KF112230	KF112754	Wu et al. 2014
*Aureoboletus gentilis*	ADK4865	Belgium	KT823961	KT824027	KT823994	Raspé et al. 2016
*Aureoboletus moravicus*	VDKO1120	Belgium	MG212528	MG212573	MG212615	This study
*Aureoboletus nephrosporus*	HKAS67931	China	–	KT990720	KT990357	Wu et al. 2016
*Aureoboletus projectellus*	AFTOL 713	U.S.A.	DQ534604*	AY879116	AY787218	Binder and Hibbett 2006*; Binder et al. unpubl.
*Aureoboletus thibetanus*	HKAS76655	China	–	KF112236	KF112752	Wu et al. 2014
*Aureoboletus tomentosus*	HKAS80485	China	–	KT990715	KT990353	Wu et al. 2016
*Aureoboletus viscosus*	HKAS53398	China	–	KF112238	KF112755	Wu et al. 2014
*Aureoboletus zangii*	HKAS74766	China	–	KT990726	KT990363	Wu et al. 2016
Austroboletus cf. dictyotus	OR045	Thailand	KT823966	KT824032	KT823999	Raspé et al. 2016
*Austroboletus olivaceoglutinosus*	HKAS57756	China	–	KF112212	KF112764	Wu et al. 2014
*Austroboletus* sp.	HKAS59624	China	–	KF112217	KF112765	Wu et al. 2014
*Baorangia pseudocalopus*	HKAS63607	China	–	KF112167	KF112677	Wu et al. 2014
*Baorangia pseudocalopus*	HKAS75739	China	–	KJ184570	KM605179	Wu et al. 2015
Boletellus aff. emodensis	OR061	Thailand	KT823970	KT824036	KT824003	Raspé et al. 2016
*Boletellus* sp.	HKAS58713	China	–	KF112307	KF112759	Wu et al. 2014
*Boletellus* sp.	HKAS59536	China	–	KF112306	KF112758	Wu et al. 2014
*Boletellus* sp.	OR0621	Thailand	MG212529	MG212574	MG212616	This study
*Boletus aereus*	VDKO1055	Belgium	MG212530	MG212575	MG212617	This study
*Boletus albobrunnescens*	OR131	Thailand	KT823973	KT824039	KT824006	Raspé et al. 2016
*Boletus botryoides*	HKAS53403	China	–	KT990738	KT990375	Wu et al. 2016
*Boletus edulis*	VDKO0869	Belgium	MG212531	MG212576	MG212618	This study
*Boletus* s.s. sp.	OR0446	China	MG212532	MG212577	MG212619	This study
*Boletus erythropus*	VDKO0690	Belgium	KT823982	KT824048	KT824015	Raspé et al. 2016
*Borofutus dhakanus*	HKAS73789	Bangladesh	–	JQ928576	JQ928597	Hosen et al. 2013
*Borofutus dhakanus*	HKAS73785	Bangladesh	–	JQ928577	JQ928596	Hosen et al. 2013
*Borofutus dhakanus*	OR345	Thailand	MG212533	MG212578	MG212620	This study
*Borofutus dhakanus*	OR352	Thailand	MG212534	MG212579	MG212621	This study
*Borofutus dhakanus*	SV210	Thailand	MG212535	MG212580	MG212622	This study
*Borofutus dhakanus*	SV245	Thailand	MG212536	MG212581	MG212623	This study
*Butyriboletus appendiculatus*	VDKO0193b	Belgium	MG212537	MG212582	MG212624	This study
*Butyriboletus pseudoregius*	VDKO0925	Belgium	MG212538	MG212583	MG212625	This study
*Butyriboletus pseudospeciosus*	HKAS63513	China	–	KT990743	KT990380	Wu et al. 2016
*Butyriboletus roseoflavus*	HKAS54099	China	–	KF739779	KF739703	Wu et al. 2014
*Butyriboletus subsplendidus*	HKAS50444	China	–	KT990742	KT990379	Wu et al. 2016
Butyroboletus cf. roseoflavus	OR230	China	KT823974	KT824040	KT824007	Raspé et al. 2016
*Caloboletus calopus*	ADK4087	Belgium	MG212539	KJ184566	KP055030	This study; Zhao et al. 2014a; Zhao et al. 2014b
*Caloboletus radicans*	VDKO1187	Belgium	MG212540	MG212584	MG212626	This study
*Caloboletus yunnanensis*	HKAS69214	China	–	KJ184568	KT990396	Zhao et al. 2014a; Wu et al. 2016
Chalciporus aff. piperatus	OR586	Thailand	KT823976	KT824042	KT824009	Raspé et al. 2016
*Chalciporus africanus*	JD517	Cameroon	KT823963	KT824029	KT823996	Raspé et al. 2016
*Chalciporus rubinus*	AF2835	Belgium	KT823962	KT824028	KT823995	Raspé et al. 2016
*Chiua virens*	OR0266	China	MG212541	MG212585	MG212627	This study
*Chiua viridula*	HKAS74928	China	–	KF112273	KF112794	Wu et al. 2014
Crocinoboletus cf. laetissimus	OR576	Thailand	KT823975	KT824041	KT824008	Raspé et al. 2016
*Cyanoboletus brunneoruber*	OR0233	China	MG212542	MG212586	MG212628	This study
*Cyanoboletus pulverulentus*	RW109	Belgium	KT823980	KT824046	KT824013	Raspé et al. 2016
*Cyanoboletus* sp.	OR0257	China	MG212543	MG212587	MG212629	This study
*Fistulinella prunicolor*	REH9502	Australia	MG212544	MG212588	MG212630	This study
*Harrya chromapes*	KPM NC17835	Japan	KC552173	JN378457	–	Orihara et al. 2016; Orihara et al. 2012
*Harrya moniliformis*	HKAS49627	China	–	KT990881	KT990500	Wu et al. 2016
Heimioporus cf. mandarinus	OR0661	Thailand	MG212545	MG212589	MG212631	This study
*Heimioporus japonicus*	OR114	Thailand	KT823971	KT824037	KT824004	Raspé et al. 2016
*Heimioporus retisporus*	HKAS52237	China	–	KF112228	KF112806	This study
*Heimioporus* sp.	OR0218	Thailand	MG212546	MG212590	MG212632	This study
*Hemileccinum depilatum*	AF2845	Belgium	MG212547	MG212591	MG212633	This study
*Hemileccinum impolitum*	ADK4078	Belgium	MG212548	MG212592	MG212634	This study
*Hemileccinum rugosum*	HKAS84970	China	–	KT990773	KT990412	Wu et al. 2016
*Hourangia cheoi*	HKAS74744	China	–	KF112285	KF112772	Wu et al. 2014
*Hourangia nigropunctata*	HKAS 57427	China	–	KP136927	KP136978	Zhu et al. 2015
*Hymenoboletus luteopurpureus*	HKAS46334	China	–	KF112271	KF112795	Wu et al. 2014
*Imleria badia*	VDKO0709	Belgium	KT823983	KT824049	KT824016	Raspé et al. 2016
*Lanmaoa angustispora*	HKAS74752	China	–	KM605154	KM605177	Wu et al. 2015
*Lanmaoa asiatica*	HKAS63603	China	–	KM605153	KM605176	Wu et al. 2015
*Leccinellum crocipodium*	VDKO1006	Belgium	KT823988	KT824054	KT824021	Raspé et al. 2016
*Leccinellum* sp.	KPM-NC-0018041	Japan	KC552165	KC552094	–	Orihara et al. 2016
*Leccinum scabrum*	VDKO0938	Belgium	MG212549	MG212593	MG212635	This study
*Leccinum scabrum*	RW105a	Belgium	KT823979	KT824045	KT824012	Raspé et al. 2016
*Leccinum scabrum*	KPM-NC-0017840	Scotland	KC552170	JN378455	–	Orihara et al. 2016; Orihara et al. 2012
*Leccinum schistophilum*	VDKO1128	Belgium	KT823989	KT824055	KT824022	Raspé et al. 2016
*Leccinum variicolor*	VDKO0844	Belgium	MG212550	MG212594	MG212636	This study
*Leccinum versipelle*	KPM-NC-0017833	Scotland	KC552172	JN378454	–	Orihara et al. 2016; Orihara et al. 2012
*Leccinum vulpinum*	KPM-NC-0017834	Scotland	KC552171	JN378456	–	Orihara et al. 2016; Orihara et al. 2012
*Mucilopilus castaneiceps*	HKAS75045	China	–	KF112211	KF112735	Wu et al. 2014
*Neoboletus brunneissimus*	HKAS50538	China	–	KM605150	KM605173	Wu et al. 2015
*Neoboletus brunneissimus*	OR0249	China	MG212551	MG212595	MG212637	This study
*Neoboletus junquilleus*	AF2922	France	MG212552	MG212596	MG212638	This study
*Neoboletus magnificus*	HKAS54096	China	–	KF112149	KF112654	Wu et al. 2014
*Neoboletus venenatus*	HKAS63535	China	–	KT990807	KT990448	Wu et al. 2016
*Octaviania asahimontana*	KPM-NC17824	Japan	KC552154	JN378430	–	Orihara et al. 2016; Orihara et al. 2012
*Octaviania asterosperma*	AQUI3899	Italy	KC552159	KC552093	–	Orihara et al. 2016
*Octaviania celatifilia*	KPM-NC17776	Japan	KC552147	JN378416	–	Orihara et al. 2016; Orihara et al. 2012
*Octaviania decimae*	KPM-NC17763	Japan	KC552145	JN378409	–	Orihara et al. 2016; Orihara et al. 2012
*Octaviania tasmanica*	MEL2341996	Australia	KC552156	JN378436	–	Orihara et al. 2016; Orihara et al. 2012
*Octaviania zelleri*	MES270	U.S.A.	KC552161	JN378440	–	Orihara et al. 2016; Orihara et al. 2012
*Phylloporus brunneiceps*	OR050	Thailand	KT823968	KT824034	KT824001	Raspé et al. 2016
*Phylloporus castanopsidis*	OR052	Thailand	KT823969	KT824035	KT824002	Raspé et al. 2016
*Phylloporus imbricatus*	HKAS68642	China	–	KF112299	KF112786	Wu et al. 2014
*Phylloporus luxiensis*	HKAS75077	China	–	KF112298	KF112785	Wu et al. 2014
*Phylloporus yunnanensis*	OR0448	China	MG212554	MG212598	MG212640	This study
*Porphyrellus castaneus*	OR0241	China	MG212555	MG212599	MG212641	This study
*Porphyrellus porphyrosporus*	MB97-023	Germany	DQ534609	GU187734	GU187800	Binder and Hibbett 2006; Binder et al. 2010
Pulveroboletus aff. ravenelii	ADK4360	Togo	KT823957	KT824023	KT823990	Raspé et al. 2016
Pulveroboletus aff. ravenelii	ADK4650	Togo	KT823959	KT824025	KT823992	Raspé et al. 2016
Pulveroboletus aff. ravenelii	HKAS53351	China	–	KF112261	KF112712	Wu et al. 2014
*Pulveroboletus fragrans*	OR673	Thailand	KT823977	KT824043	KT824010	Raspé et al. 2016
*Pulveroboletus ravenelii*	REH2565	U.S.A.	KU665635	KU665636	KU665637	Raspé et al. 2016
*Pulveroboletus* sp.	HKAS74933	China	–	KF112262	KF112713	Wu et al. 2014
Retiboletus aff. nigerrimus	OR049	Thailand	KT823967	KT824033	KT824000	Raspé et al. 2016
*Retiboletus fuscus*	OR0231	China	MG212556	MG212600	MG212642	This study
*Retiboletus griseus*	MB03-079	U.S.A.	KT823964	KT824030	KT823997	Raspé et al. 2016
*Retiboletus kauffmanii*	OR0278	China	MG212557	MG212601	MG212643	This study
*Retiboletus nigerrimus*	HKAS53418	China	–	KT990824	KT990462	Wu et al. 2016
*Retiboletus sinensis*	HKAS59832	China	–	KT990827	KT990464	Wu et al. 2016
*Rhodactina himalayensis*	CMU25117	Thailand	MG212558	MG212602, MG212603	–	This study
*Rhodactina incarnata*	CMU25116	Thailand	DQ328982	–	–	Yang et al. 2006
*Rhodactina rostratispora*	OR1055	Thailand	MG212559	MG212604	MG212644	This study
*Rhodactina rostratispora*	SV170	Thailand	MG212560	MG212605	MG212645	This study
*Rhodactina rostratispora*	SV208	Thailand	MG212561	MG212606	MG212646	This study
*Rossbeevera cryptocyanea*	KPM-NC17843	Japan	KT581441	KC552072	–	Orihara et al. 2016
*Rossbeevera eucyanea*	TNS-F-36986	Japan	KC552115	KC552068	–	Orihara et al. 2016
*Rossbeevera griseovelutina*	TNS-F-36989	Japan	KC552124	KC552076	–	Orihara et al. 2016
*Rossbeevera pachydermis*	KPM-NC23336	New Zealand	KJ001064	KP222912	–	Orihara et al. 2016
*Rossbeevera vittatispora*	TO-AUS-72	Australia	KC552108	KC552065	–	Orihara et al. 2016
*Royoungia reticulata*	HKAS52253	China	–	KT990786	KT990427	Wu et al. 2016
*Royoungia rubina*	HKAS53379	China	–	KF112274	KF112796	Wu et al. 2014
*Rubroboletus legaliae*	VDKO0936	Belgium	KT823985	KT824051	KT824018	Raspé et al. 2016
*Rubroboletus satanas*	VDKO0968	Belgium	KT823986	KT824052	KT824019	Raspé et al. 2016
*Rubroboletus sinicus*	HKAS56304	China	–	KJ619483	KP055031	Zhao et al. 2014a; Zhao et al. 2014b
*Rugiboletus brunneiporus*	HKAS83209	China	–	KM605144	KM605168	Wu et al. 2015
*Rugiboletus extremiorientalis*	HKAS76663	China	–	KM605147	KM605170	Wu et al. 2015
*Rugiboletus extremiorientalis*	OR0406	Thailand	MG212562	MG212607	MG212647	This study
*Spongiforma thailandica*	DED7873	Thailand	MG212563	KF030436*	MG212648	Nuhn et al. 2013*; This study
*Strobilomyces atrosquamosus*	HKAS55368	China	–	KT990839	KT990476	Wu et al. 2016
*Strobilomyces echinocephalus*	OR0243	China	MG212564	MG212608	MG212649	This study
*Strobilomyces floccopus*	RW103	Belgium	KT823978	KT824044	KT824011	Raspé et al. 2016
*Strobilomyces mirandus*	OR115	Thailand	KT823972	KT824038	KT824005	Raspé et al. 2016
*Strobilomyces* sp.	OR0259	China	MG212565	MG212609	MG212650	This study
*Strobilomyces* sp.	OR0778	Thailand	MG212566	MG212610	MG212651	This study
*Strobilomyces verruculosus*	HKAS55389	China	–	KF112259	KF112813	Wu et al. 2014
*Suillellus luridus*	VDKO0241b	Belgium	KT823981	KT824047	KT824014	Raspé et al. 2016
*Suillellus subamygdalinus*	HKAS53641	China	–	KT990841	KT990478	Wu et al. 2016
*Sutorius australiensis*	REH9441	Australia	MG212567	JQ327032*	MG212652	Halling et al. 2012*; This study
*Sutorius eximius*	REH9400	U.S.A.	MG212568	JQ327029*	MG212653	Halling et al. 2012*; This study
*Turmalinea persicina*	KPM-NC18001	Japan	KC552130	KC552082	–	Orihara et al. 2016
*Turmalinea yuwanensis*	KPM-NC18011	Japan	KC552138	KC552089	–	Orihara et al. 2016
*Tylocinum griseolum*	HKAS50281	China	–	KF112284	KF112730	Wu et al. 2014
*Tylopilus atripurpureus*	HKAS50208	China	–	KF112283	KF112799	Wu et al. 2014
*Tylopilus balloui* s.l.	OR039	Thailand	KT823965	KT824031	KT823998	Raspé et al. 2016
*Tylopilus felleus*	VDKO0992	Belgium	KT823987	KT824053	KT824020	Raspé et al. 2016
*Tylopilus* sp.	OR0252	China	MG212569	MG212611	MG212654	This study
*Tylopilus* sp.	OR0542	Thailand	MG212570	MG212612	MG212655	This study
*Tylopilus vinaceipallidus*	OR0137	China	MG212571	MG212613	MG212656	This study
*Veloporphyrellus alpinus*	HKAS57490	China	JX984514	JX984549	–	Li et al. 2014
*Veloporphyrellus conicus*	CFMR BZ1670	Belize	JX984520	JX984555	–	Li et al. 2014
*Veloporphyrellus pseudovelatus*	HKAS52258	China	JX984517	JX984551	–	Li et al. 2014
*Veloporphyrellus velatus*	HKAS63668	China	JX984523	JX984554	–	Li et al. 2014
*Xerocomellus chrysenteron*	VDKO0821	Belgium	KT823984	KT824050	KT824017	Raspé et al. 2016
*Xerocomellus cisalpinus*	ADK4864	Belgium	KT823960	KT824026	KT823993	Raspé et al. 2016
*Xerocomus fulvipes*	HKAS76666	China	–	KF112292	KF112789	Wu et al. 2014
*Xerocomus subtomentosus*	VDKO0987	Belgium	MG212572	MG212614	MG212657	This study
*Zangia citrina*	HKAS52684	China	HQ326850	HQ326872	–	Li et al. 2011
*Zangia olivacea*	HKAS55830	China	HQ326855	HQ326874	–	Li et al. 2011
*Zangia olivaceobrunnea*	HKAS52275	China	HQ326856	HQ326875	–	Li et al. 2011
*Zangia roseola*	HKAS51137	China	HQ326858	HQ326877	–	Li et al. 2011

## Taxonomy

### Key to the species of *Rhodactina*

**Table:** 

1	Basidiospores with a markedly prominent hilar appendage 2.5–5 µm long and 3.5–5 µm wide with a terminal hilum, spore size 12–16 × 10–14 µm	***R. rostratispora* sp. nov.**
–	Basidiospores without markedly prominent hilar appendage or with short to nearly truncate hilar appendage up to 1.5 µm long and 1.5 µm wide	**2**
2	Basidiospores bearing large (5)6–7(8) longitudinal ridges, 3–4 µm wide, up to 5 µm tall, dark violet in 5 % KOH, spore size 15–20 × 12.5–18 µm	***R. himalayensis***
–	Basidiospores bearing (7)8–9(10) longitudinal ridges, 2–3 µm wide, up to 3 µm tall, slightly reddish to purplish yellow in 5 % KOH, spore size 10–13 × 10–12 µm	***R. incarnata***

#### Rhodactina rostratispora

Taxon classificationFungiBoletalesBoletaceae

Vadthanarat, Raspé & Lumyong sp. nov.

822126

Figs 2 , 3 , 4

##### Type.

THAILAND, Ubon Ratchathani Province, Trakan Phuet Phon District, Don Khok Tam Lae community forest, 15°35'46"N, 105°06'38"E, elev. 150 m., 28 July 2015, S. Vadthanarat 170, (holotype: CMUB!; isotype: BR!).

##### Etymology.

From Latin “rostrati–” meaning having beaked prow or a solid projection and “spora” meaning spores, referring to the basidiospores having a markedly prominent and large hilar appendage.

##### Description.

*Basidiomata* small to medium-sized 0.8–2.5(4.5) cm diam., subglobose to ovoid with a rudimentary elongated basal attachment, with greyish white to pale brown rhizoids at the base and going up along the surface of basidiomata to about half of the height. *Peridium surface* (outer peridium) fibrillose to arachnoid, off-white to pinkish white (7A2–3 to 9A2), dull, moist, cracked in places. *Peridium* very thin, 0.1–0.2(0.4) mm thick. *Hymenophore* cartilaginous, completely enclosed, whitish orange to reddish orange (7A3–4 to 8A5–6) at first becoming orangey red to red (9D–E8 to 10D–E8) with age, then dark red when very old, irregular; *Stipe-columella* absent. *Taste* fungoid. *Odour* absent when young, very strongly fruity alcoholic when old.

*Macrochemical reactions*: hymenophore turned dark purplish (15F8) to greyish violet (19D3) with 5% KOH, slightly greyish violet (19D3) with NH_4_OH.

*Basidiospores* [404/8/8] (11.5–)12–13.6–15(–16) × (10–)10.5–11.7–13(–14), *Q* = (1–)1.04–1.16–1.3(–1.4), from the holotype, (12–)12–13.5–15.2(–16) × (10–)10–11.6–13.2(–14) µm, *Q* = (1–)1–1.02–1.33(–1.4), *N* = 50, ellipsoid to broadly ellipsoid with longitudinal ridges, stellate in polar-view, thick-walled (1–1.5 µm thick), yellowish to orangey hyaline to reddish yellow at first, reddish to brownish yellow with age in water, slightly purplish and slightly more reddish to brownish in 5% KOH, slightly purplish hyaline in NH_4_OH, slightly dextrinoid to dextrinoid in Melzer’s reagent; ornamentation (7)8–9 solid ridges regularly and longitudinally arranged under light microscope, sometimes anastomosing under SEM, 2–3 µm tall and 2–2.5 µm wide at the base; hilar appendage prominent, 2.5–5 µm long with a terminal hilum. *Basidia* 4–spored, (26–)26.1–32.3–36(–36) × (8–)8–9.5–11(–11) µm (*n* = 20; from holotype only), clavate to cylindrical, hyaline in water, 5% KOH and NH_4_OH, yellowish hyaline in Melzer’s reagent; sterigmata broken by spore release, stout, 3–4 µm long. *Cystidia* none observed. *Hymenophoral trama* 60–130 µm thick, irregular, with a narrow, central layer of subparallel to loosely interwoven, 3–7(8) µm wide, thin-walled hyphae, slightly gelatinised, hyaline in water, 5% KOH and NH_4_OH. *Peridiopellis* a tomentum 45–120 µm thick, poorly differentiated, composed of thin-walled, 3–10 µm wide hyphae, anastomosing at places and covered with yellowish brown incrustations on the surface at places, otherwise hyaline in water, 5% KOH and NH_4_OH, inamyloid. *Clamp connections* not seen in any of the tissues.

**Figure 2.**
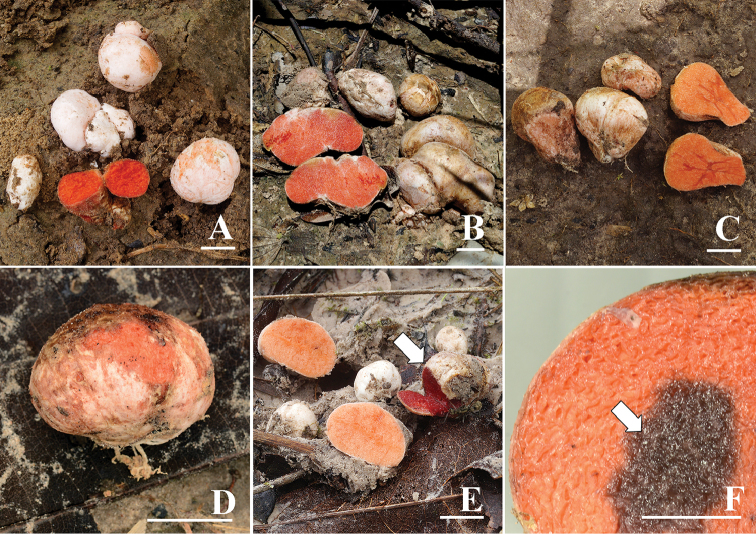
Basidiomata of *Rhodactina rostratispora* **A** S. Vadthanarat 170 (holotype) **B** S. Vadthanarat 206 **C** S. Vadthanarat 208 **D** O. Raspé 1055 **E** S. Vadthanarat 406, showing one basidioma (white arrow) that had a strong fruity alcoholic smell **F** Hymenophore turned dark purple to greyish violet with 5% KOH (white arrow). Scale bars: **A–E** = 1 cm; **F** =0.5 cm.

**Figure 3.**
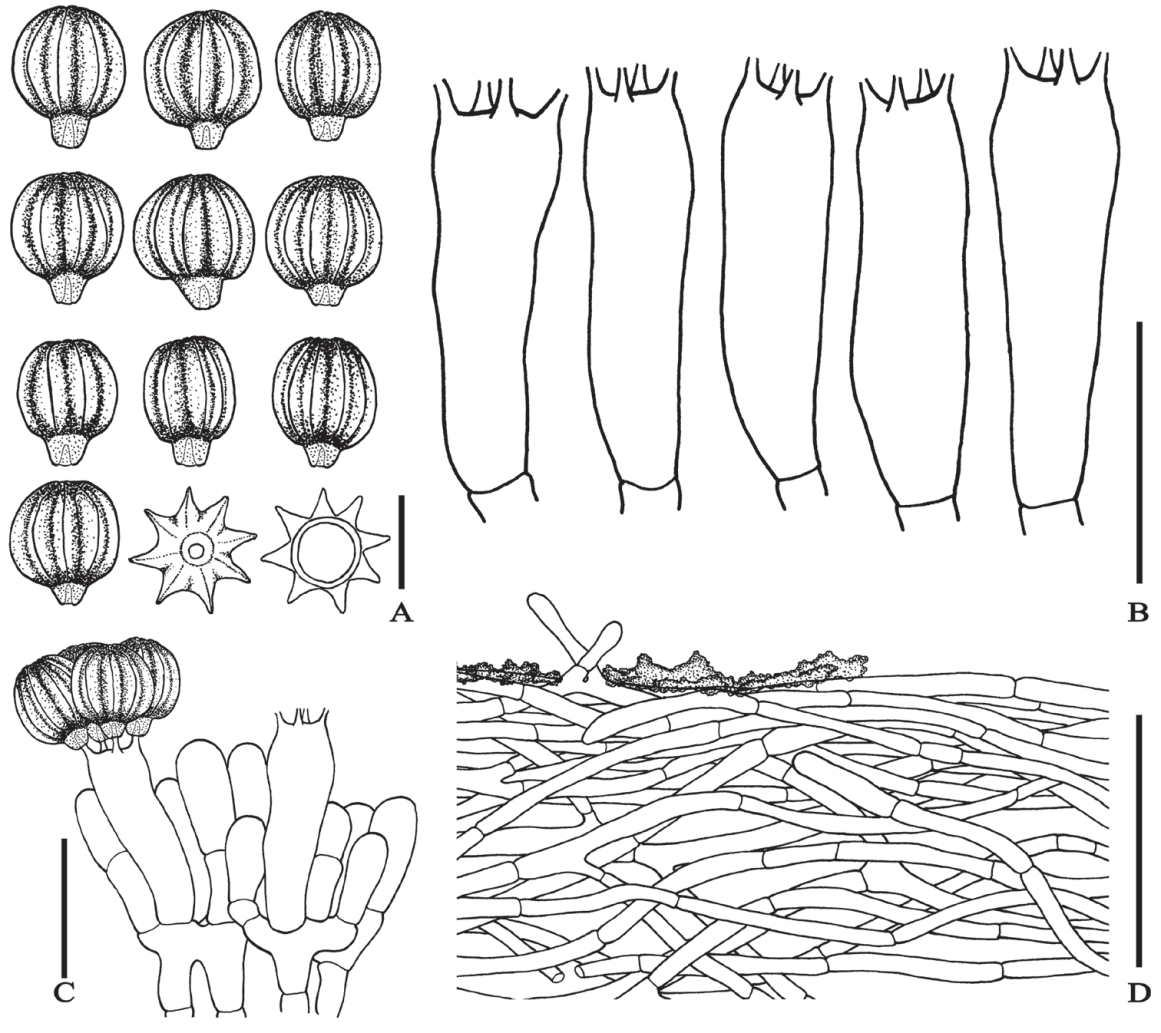
Microscopic features of *Rhodactina rostratispora* **A** Basidiospores in side view, polar view and optical section **B** Basidia **C** Hymenium showing basidia and basidioles **D** Peridiopellis covered with some encrustations. All drawings were made from the type. Scale bars: **A** = 10 µm; **B–C** = 20 µm; **D** = 50 µm.

**Figure 4.**
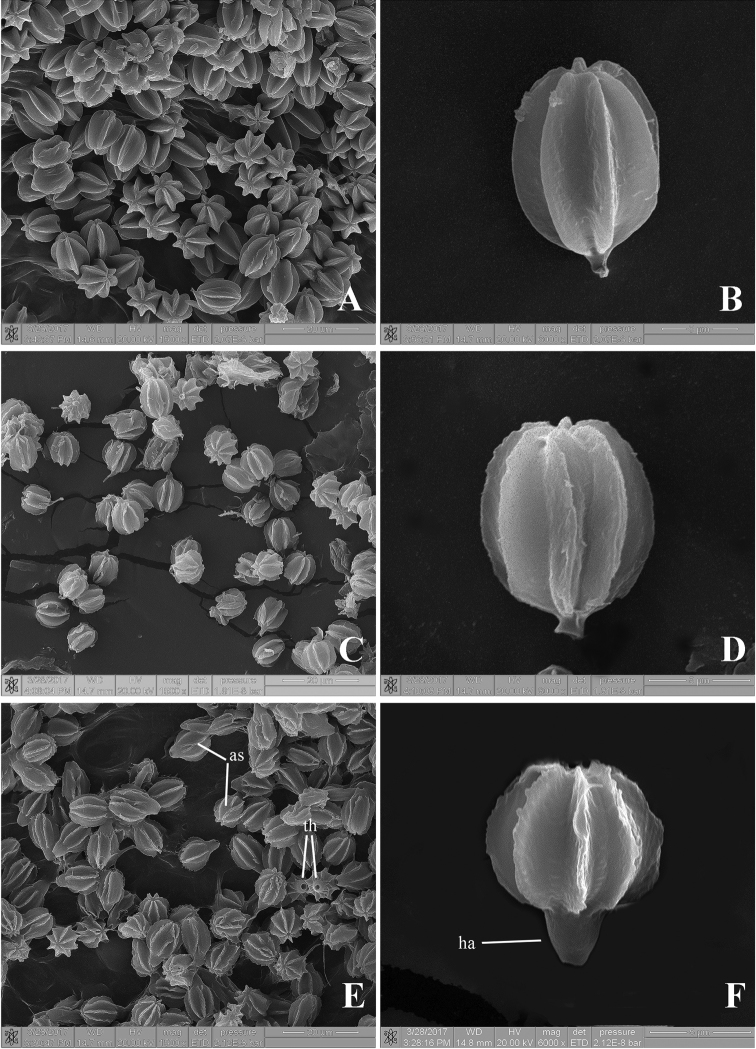
Scanning electron micrographs of basidiospores **A–B** *Rhodactina himalayensis* (CMU25117) showing the basidiospores with 6–7 longitudinal ridges **C–D** *Rhodactina incarnata* (CMU25116, holotype) showing the basidiospores with 8–9 longitudinal ridges **E–F** *Rhodactina rostratispora* (O. Raspé 1055) showing the basidiospores with 8–9 longitudinal ridges, the wide and prominent hilar appendage (ha), a terminal hilum (th) and anastomosing ridges in some spores (as).

##### Habit and habitat.

Subepigeal, solitary to gregarious (4–7 basidiomata), or fasciculate by 2–5 basidiomata, on sandy soil in dipterocarp forest dominated by *Dipterocarpus tuberculatus*, *D. intricatus*, *D. obtusifolius*, *Shorea obtusa*, *S. siamensis* and *Eucalyptus* sp.

##### Known distribution.

Currently found only from Ubon Ratchathani province, northeastern Thailand.

##### Additional specimens examined.

*Rhodactina rostratispora*.—THAILAND, Ubon Ratchathani Province, Trakan Phuet Phon District, Don Khok Tam Lae community forest, 15°35'40.2"N–105°06'37.8"E, elev. 150 m., 28 July 2015, S. Vadthanarat 169, (CMUB, BR); ibid. 15°35'41.5"N–105°06'35.4"E, elev. 150 m., 28 July 2015, O. Raspé 1055, (CMUB, BR); ibid. 15°35'48.3"N –105°06'35.9"E, elev. 150 m., 6 August 2015, S. Vadthanarat 206, (CMUB, BR); ibid. 15°35'52.4"N–105°06'41.2"E, elev. 150 m., 6 August 2015, S. Vadthanarat 208, (CMUB, BR); ibid. 15°35'56.1"N–105°06'38.9"E, elev. 150 m., 6 August 2015, S. Vadthanarat 212, (CMUB, BR); ibid. 15°36'2.6"N–105°06'36.7"E, elev. 150 m., 14 May 2017, S. Vadthanarat 376, (CMUB, BR); Ban Huay Fai community forest, 15°32'42.7"N–105°10'16.3"E, elev. 160 m., 15 July 2017, S. Vadthanarat 406, (CMUB, BR).

*R. himalayensis*. – THAILAND, Chiang Mai Province, Doi Suthep-Pui National Park, forest behind Channel 9 TV station, 4 August 2000, Saisamorn Lumyong, Pipob Lumyong, Rarunee Sanmee and B. Dell 2254 (CMU25117).

*R. incarnata*. – THAILAND, Chiang Mai Province, Sanpatong District, Mae Wang, Conservation forest, Sanpatong-Ban Guard Rd., 24 July 2002, Saisamorn Lumyong, Pipob Lumyong, Rarunee Sanmee and Zhu L. Yang 45209 (CMU25116; holotype).

##### Remarks.

*Rhodactina rostratispora* is characterised by its basidiospores having a markedly prominent hilar appendage (2.5–5 µm long, 3.5–5 µm wide), with a terminal hilum; ornamentation consisting of (7)8–9 longitudinal ridges, and (11.5–)12–13.6–15(–16) × (10–)10.5–11.7–13(–14) µm. *R. himalayensis* has larger basidiospores (15–20 × 12.5–18 µm) without prominent hilar appendage, with fewer [(5)6–7(8)], broader ridges, while *R. incarnata* has a similar spore size (10–13 × 10–12 µm) and the same number of spore ridges [(7)8–9(10)] as the new species, but it does not have the prominent hilar appendage.

In one *R. rostratispora* specimen (S. Vadthanarat 208), abnormal spores were observed. Those spores were elongated, 21–24 × 4–8 µm, thick-walled, narrowly fusiform to bacilliform, with or without longitudinal ridges, more or less constricted in the middle. They were usually found attached to apparently normal basidia with four sterigmata.

## Discussion

Morphologically, the new species *R. rostratispora* is characterised by its ridged basidiospores having a markedly prominent hilar appendage with a terminal hilum, which is not found in other *Rhodactina* species (Pegler and Young 1989, Yang et al. 2006). However, ridged basidiospores having a prominent hilar appendage are found in some other sequestrate Boletaceae in the genus *Turmalinea* Orihara & N. Maek and *Rossbeevera*, including *T. persicina* Orihara, *T. chrysocarpa* Orihara & Z.W. Ge, *T. mesomorpha* Orihara, *Ro. paracyanea* Orihara and *Ro. cryptocyanea* Orihara. The basidiospores of those species have a long pointed hilar appendage 4.5–6 µm (Orihara et al. 2016) but are not as wide as in *R. rostratispora* (2.5–5 µm long, 3.5–5 µm wide) and also their hilar appendage lacks a terminal hilum. Macroscopically, those species differ from *R. rostratispora* in that both *Rossbeevera* and *Turmalinea* have basidiomata often turning blue to greenish blue when bruised, which has never been reported in any *Rhodactina* species (Pegler and Young 1989, Yang et al. 2006). Moreover, the colour of mature hymenophore of *Turmalinea* and *Rossbeevera* species are dark brown or blackish brown (Lebel et al. 2012, Orihara et al. 2016) not red or dark red like in *Rhodactina*.

The phylogenetic analyses also support the placement of the new taxon in the genus *Rhodactina*, with *R. incarnata* being the closest species. The phylogenetic tree also showed that *Rhodactina* is sister to a clade composed of *Spongiforma* and *Borofutus* within the subfamily Leccinoideae, with 100% bootstrap support. According to Wu et al. (2016), there are 10 genera in the sub-family Leccinoideae including *Borofutus*, *Chamonixia* Rolland, *Leccinum* Gray, *Leccinellum* Bresinsky & Manfr. Binder, *Octaviania* Vittad, *Pseudoaustroboletus* Y.C. Li & Zhu L. Yang, *Retiboletus* Manfr. Binder & Bresinsky, *Rossbeevera* T. Lebel & Orihara & N. Maek, *Spongiforma* and *Tylocinum* Yan C. Li & Zhu L. Yang. The phylogenetic analyses infer that *Rhodactina* is the eleventh genus in the subfamily.

In the examination of *R. rostratispora*, it was found that the hymenophore turned dark purplish to greyish violet with 5% KOH. Interestingly, all of the genera in subfamily Leccinoideae that turn purple to violet with aqueous KOH solution, namely *Rhodactina*, *Borofutus* and *Spongiforma*, are grouped in one clade with 100% bootstrap support. All of the species in the clade share the characteristic of the basidiospores turning more or less purplish, purplish red to violet grey in aqueous KOH solution (Desjardin et al. 2009, Hosen et al. 2013). *Spongiforma squarepantsii* Desjardin, Peay & T.D. Bruns, which was described from Malaysia, was not included in these analyses, but the original description of this species also mentioned that its basidiospores turn pale lilac grey in 3% KOH (Desjardin et al. 2011). A chemical reaction with KOH was observed not only with basidiospores, but also on the hymenophore (Desjardin et al. 2009). The reaction to 5% KOH has been observed on fresh basidiomata of *Borofutus dhakanus* Hosen & Zhu L. Yang which is an epigeous species and the only currently known species of this genus. The colour reaction of pileus and pileus context, which turned pinkish blue to purplish blue, was different from that of the stipe and stipe context, which turned yellowish green to olive green. This variation in colour of the reaction to 5% KOH was not mentioned in the original description of the species (Hosen et al. 2013). Therefore, this chemical character is very useful for the identification of boletes belonging to this group. Other taxa that have been reported to show similar colour reactions to KOH and would, therefore, belong to this group, include *Austroboletus longipes* (Massee) Wolfe, *Austroboletus malaccensis* (Pat. & C.F. Baker) Wolfe and *Austroboletus tristis* (Pat. & C.F. Baker) Wolfe (Corner 1972, Horak 2011).

Some basidiomata of *R. rostratispora* were old when collected, with dark red hymenophore and had a very strong fruity, alcoholic odour. The odour seems to be present in old basidiomata only (S. Vadthanarat 212 and one basidiomata of S. Vadthanarat 406). One possible explanation to the alcoholic smell is that sterigmata are broken from spore release and any remaining cytoplasm in the basidia could leak into the cavities of the hymenophore and be fermented. Fermentation by yeasts might be possible due to the cracking of the peridium, allowing contact of the hymenophore cavities with ambient air. As mammals and marsupials are known to be the main spore dispersal vectors of truffle-like fungi (e.g. Lamont et al. 1985, Cázares and Trappe 1994, Vernes and Dunn 2009), the strong alcoholic smell could facilitate detection and entice consumption of the basidiomata by mammals and thus help spore dispersal.

The three *Rhodactina* species were found only in dipterocarp forest between 100 to 600 m above sea level in India, northern and northeastern Thailand (Pegler and Young 1989, Yang et al. 2006). They presumably form ectomycorrhizal associations with trees of the genera *Dipterocarpus* and *Shorea* (Dipterocarpaceae). However, in the forest where the new species was found, some scattered *Eucalyptus* trees were also observed. As *Eucalyptus* species have been reported to be ectomycorrhizal trees (e.g. Giachini et al. 2000, Ducousso et al. 2012, Garrett Kluthe et al. 2016), the *Eucalyptus* trees found in the forest could also possibly be host of *R. rostratispora*. However, *Eucalyptus* is not indigenous to Thailand; several species have been planted since the early 1900s (Luangviriyasaeng 2003). As *Rhodactina* species seem to be indigenous to Thailand and *Eucalyptus* not, they are less likely to be ectomycorrhizal partners. Further study is needed, however, to confirm the range of ectomycorrhizal host tree species of *R. rostratispora*. *Borofutus* and *Spongiforma*, the most closely related genera of *Rhodactina*, are also ectomycorrhizal associates with trees in Dipterocarpaceae. The only known *Borofutus* species, *B. dhakanus* is ectomycorrhizal with *Shorea robusta* (Hosen et al. 2013). As for *Spongiforma* species, *S. thailandica* was reported as associated with *Dipterocarpus* sp. and *Shorea* sp. in primary forest while *S. squarepantsii* was reported as associated with unidentified dipterocarp trees (Desjardin et al. 2009, Desjardin et al. 2011).

## Supplementary Material

XML Treatment for Rhodactina rostratispora
